# Associations of Healthy Eating Behavior with Mental Health and Health-Related Quality of Life: Results from the Korean National Representative Survey

**DOI:** 10.3390/nu15245111

**Published:** 2023-12-14

**Authors:** Min-Ju Kim, Jong Eun Park, Jong Hyock Park

**Affiliations:** 1 Department of Biomedical Convergence, Chungbuk National University Graduate School, Cheongju 28644, Republic of Korea; mjk0129@gmail.com; 2 Division of Cardiovascular Disease Research, Department of Chronic Disease Convergence, Korea National Institute of Health, Cheongju 28159, Republic of Korea; 3 Institute of Health & Science Convergence, Chungbuk National University, Cheongju 28644, Republic of Korea; je.park0525@gmail.com; 4 College of Medicine, Chungbuk National University, Cheongju 28644, Republic of Korea; 5 Department of Public Health and Preventive Medicine, Chungbuk National University Hospital, Cheongju 28644, Republic of Korea

**Keywords:** Korean healthy eating index, mental health, depression, health-related quality of life, EQ-5D

## Abstract

Background: Healthy eating behaviors may be related to mental health and health-related quality of life. This study investigates the associations between diet quality, mental health, and health-related quality of life among men and women in Korea. Methods: A total of 6823 participants, aged 19, from the 7th Korea National Health and Nutrition Examination Survey from 2016 to 2018 were included. Their overall diet quality was estimated using the Korean Healthy Eating Index (KHEI). Multivariate logistic regression was used to identify the associations between diet quality, mental health, and quality of life. Results: The prevalence of stress perception and depression was highest in the lowest tertile of the KHEI score and higher for women than men. Among men, a significant association was observed only for stress perception and depressive symptoms in the second tertile, with odds ratios of 0.745 (95% CI, 0.585–0.949) and 0.519 (95% CI, 0.275–0.980). In contrast, the multivariate-adjusted odds ratios for stress perception, depressive symptoms, and low EQ-5D index among women in the highest tertile were 0.668 (95% CI, 0.541–0.823), 0.464 (95% CI, 0.288–0.746), and 0.722 (95% CI, 0.566–0.920), and significant dose–response associations were observed. Conclusions: A higher-quality diet was associated with a lower risk of stress and depression and a better quality of life. Thus, healthy eating behaviors may improve mental health and quality of life.

## 1. Introduction

Research interest in the impact of nutrition on mental health and well-being has been growing, and a poor diet may be the leading risk factor for poorer mental health [1,2]. According to the Korea Health Statistics 2020, the prevalence of stress perception and depressive symptoms was 31.5% and 5.7%, respectively [3]. The mechanisms linking diet and mental health are complex and likely bidirectional, and carbohydrate consumption, immune activation, and the gut microbiome are possible mediators [1].

Epidemiological studies have shown that healthy eating behaviors are associated with mental health, such as stress and depression symptoms, and health-related quality of life [4,5,6].

A population-based study reported that participants with a low-quality diet were 39% more likely to have depression than those with a high-quality diet [7]. In addition, a systematic review and meta-analysis showed a significant negative relationship between perceived stress and diet quality [8].

Quality of life is also likely influenced by diet and nutrition, and several studies have found that healthy dietary patterns are associated with a better health status and quality of life [9]. A longitudinal study indicated that a better quality of diet was associated with a better health-related quality of life regarding physical function, general health, energy, and emotional well-being [6]. 

Dietary habits are an important risk factor for disease prevention and have an impact on global health problems, and several previous studies have provided epidemiological evidence regarding the risk diet quality poses to mental health and quality of life [5,6,8,9]. Previous studies have mostly focused on whether dietary patterns or specific dietary components affect mental health and quality of life, but studies using an eating index for evaluating the quality of the overall diet were somewhat limited. Moreover, this study is meaningful in that it used the Korean healthy eating index developed to evaluate overall dietary habits. Recent studies in Korean adults have examined the association between diet quality and mental health; however, screening tools have not been used to measure depressive symptoms or target certain age groups [10,11]. Furthermore, few studies have reported the relationship between a healthy eating index and health-related quality of life.

In this study, we investigated the association between diet quality—evaluated using the Korean Healthy Eating Index (KHEI)—mental health, and health-related quality of life among men and women in the Republic of Korea. 

## 2. Materials and Methods

### 2.1. Study Participants

The Korea National Health and Nutrition Examination Survey (KNHANES) is a nationwide health and nutrition survey that has been conducted annually by the Korea Disease Control and Prevention Agency since 1998. This survey uses a multi-staged, stratified, rolling-sampling method based on age, sex, and geographic area to select representative households of the Korean population. Trained medical staff or interviewers conducted face-to-face interviews with a structured questionnaire. Additional details regarding KNHANES are described elsewhere [12]. A total of 24,269 respondents had participated in the 7th KNHANES from 2016 to 2018. We selected 10,076 subjects aged 19 years or older, after excluding 9313 with missing data on the Korean Healthy Eating Index (KHEI), question on stress perception, Patient Health Questionnaire-9 (PHQ-9), and Euro Quality of Life Five-Dimensions (EQ-5D) questionnaire. Of these, 180 subjects with abnormal energy intake (<500 kcal/day or >5000 kcal/day), 2517 subjects on diet therapy for disease or weight management, and 556 subjects diagnosed with stroke and cancer and with missing data on covariates were excluded from this analysis. Thus, 6823 participants (3004 men and 3819 women) were included in this study.

### 2.2. KHEI

The dietary intake data were collected by a trained nutritionist using a 24 h dietary recall. The KHEI scores were calculated using 1-day 24 h recall data. The KHEI was developed to evaluate overall diet quality and consists of 14 components in three classifications [12]: adequacy components (breakfast, mixed grain intake, total fruit intake, fresh fruit intake, total vegetable intake, vegetable intake excluding kimchi and pickled vegetables, meat/fish/egg and bean intake, milk and dairy products intake); three moderation components (percentage of energy intake from saturated fatty acid, sodium intake, and percentage of energy from sweets and beverages); and three balance components (percentage of energy intake from carbohydrates, percentage of energy intake from fat, and adequate energy intake). Each component was scored out of 10 points, but mixed grains, fruits, vegetables, and energy intake in the balance category were allotted 5 points. The KHEI score ranged from 0 to 100 points, with a higher score indicating a healthier diet. 

### 2.3. Mental Health

Stress perception was evaluated by asking about the usual level of stress experienced as follows: how much stress do you feel in your daily life? The subjects who answered extremely stressed or quite stressed were defined as having perceived stress. The Patient Health Questionnaire-9 (PHQ-9) was used to measure depressive symptoms, and participants were asked how often they had experienced each of the nine symptoms during the previous 2 weeks [13]. Each item was scored from 0 to 3, and the total PHQ-9 score ranged from 0 to 27. Participants were considered to have depression if their PHQ-9 total score was 10 or higher.

### 2.4. Health-Related Quality of Life

Health-related quality of life was assessed using the Euro Quality of Life Five-Dimensions (EQ-5D) questionnaire [14], which consisted of five dimensions: mobility, self-care, usual activities, pain/discomfort, and anxiety/depression. Each dimension was divided into three levels: no problems, some problems, and serious problems. The EQ-5D index score was converted according to a weighted formula for the Korean population, which ranged from −1 (worst health) to 1 (perfect health) [15].

### 2.5. Other Variables

The body mass index (BMI) was calculated as body weight (kg) divided by the square of the height (m^2^). Household income was categorized as low, middle-low, middle-high, or high, and the education level as elementary school or lower, middle school, high school, and college or higher. Marital status was classified as married or unmarried. Participants were considered regular drinkers if they consumed alcohol more than once per month during the previous year. Smoking status was defined as none, ex-smoker, or current smoker. Aerobic physical activity was defined as more than 2 h 30 min per week of moderate physical activity, 1 h 15 min per week of vigorous physical activity, or a combination of both.

### 2.6. Statistical Analysis

All data were analyzed using SPSS Statistics version 29 (SPSS Inc., IBM Corp., Chicago, IL, USA). The KNHANES used a two-stage stratified cluster sampling design. Complex sample analysis was performed using sampling weights according to the KNHANES analytical guidelines.

The baseline characteristics of the study participants were expressed as means ± standard error for continuous variables and number (%) for categorical variables. The subjects were divided into tertiles according to their KHEI score, and statistical differences between groups were tested using the general linear model and chi-square test. A multivariate logistic regression model was used to identify the associations between KHEI score, mental health, and quality of life with odds ratios (ORs) and 95% confidence intervals (CIs). Adjustments were made for age, BMI, household income, education, marital status, smoking status, alcohol consumption, aerobic physical activity, and total energy intake. All statistical analyses were performed separately for men and women. The *p* for trend was estimated as entering each category as a continuous variable in the multivariate logistic regression model. *p*-values of <0.05 indicated statistical significance.

## 3. Results

### 3.1. Baseline Characteristics

Table 1 shows the baseline characteristics of all study participants according to the tertiles of the KHEI score. As the KHEI score increased, both men and women were more likely to be older and married and to have better health behaviors in terms of alcohol consumption and smoking. Among men, the higher the KHEI score, the higher the level of income and education and the lower the BMI. In contrast, women with lower KHEI scores had higher levels of education and a lower total energy intake.

### 3.2. Distribution of KHEI Components According to Tertiles of the KHEI Score 

Table 2 shows the mean scores for the total and KHEI components according to the tertiles of the KHEI score. The average total KHEI score was 60.69 ± 0.31 in men and 63.62 ± 0.28 in women (*p*-value < 0.001). In both genders, all KHEI components’ scores significantly increased according to the tertiles of their KHEI score, except for sodium intake in the moderation components.

### 3.3. Mental Health and Health-Related Quality of Life According to Tertiles of the KHEI Score

Table 3 presents the prevalence of stress perception, depressive symptoms, and the EQ-5D index according to the tertiles of the KHEI score. In both men and women, the prevalence of stress and depression was highest in the lowest tertile of the KHEI score, with 30.3% and 4.2% for men and 35.1% and 8.4% for women, respectively. Additionally, women in the lowest tertile of the KHEI score had a higher prevalence of low EQ-5D scores. However, the EQ-5D index score and prevalence of a low EQ-5D index was similar across tertiles of the KHEI score. No substantial difference was observed in the EQ-5D index score and the prevalence of a low EQ-5D index by tertile of the KHEI score. Among the five EQ-5D dimensional problems, the prevalence of pain/discomfort and anxiety/depression differed significantly in women as their KHEI score increased.

### 3.4. Association between KHEI Score, Mental Health, and Health-Related Quality of Life

Table 4 shows the results of the multivariate logistic regression analysis of stress perception, depressive symptoms, and low EQ-5D index by KHEI score. For men, after adjusting for potential confounding variables, a significant association was observed only for stress perception and depressive symptoms in the second tertile, with odds ratios of 0.745 (95% CI, 0.585–0.949) and 0.519 (95% CI, 0.275–0.980), respectively. However, no significant association was observed between the tertiles of the KHEI score and a low EQ-5D index. In contrast, when compared to women in the lowest tertile of the KHEI score, the multivariate-adjusted odds ratios for stress perception, depressive symptoms, and a low EQ-5D index score in women in the highest tertile were 0.668 (95% CI, 0.541–0.823), 0.464 (95% CI, 0.288–0.746), and 0.722 (95% CI, 0.566–0.920), respectively, and significant dose–response associations were observed (*p* for trend <0.05). In addition, the highest tertile of the KHEI score was significantly associated with more mobility problems and anxiety/depression, with odds ratios of 0.586 (95% CI, 0.427–0.805) and 0.663 (95% CI, 0.484–0.910), respectively [Table S1]. After further analysis, as participants were divided into age brackets of 19–64 and 65–80 years old, only women aged 19–64 years in the highest tertile of the KHEI score had a decreased risk of stress perception, depressive symptoms, and better health-related quality of life [Table S2].

## 4. Discussion

This study aimed to examine the relationships between diet quality, mental health, and health-related quality of life in Korean adults. Although men showed a significant association between mental health and the second KHEI tertile, this association was more pronounced among women. We found that the risk of stress perception and depressive symptoms in women exhibited decreasing trends in relation to the tertiles of the KHEI score. Moreover, women with a better diet quality were more likely to have a better quality of life, with additional associations with mobility and anxiety/depression problems after adjusting for potential confounders. 

The KHEI consists of 14 components based on the Korean Dietary Guidelines and is used to assess an individual’s overall diet quality [12]. Similar to the KHEI, the United States (US) HEI was developed for the US population, and the total HEI scores of US adults were 57.2 for men and 59.7 for women [16]. Our results also showed that the total KHEI score was lower in men than in women (60.7 vs. 63.6). Women displayed higher scores for breakfast, mixed grains, fruits, milk and dairy, saturated fatty acids, sodium, sweets and beverages, while the scores for vegetables, meat/fish/eggs and legumes, carbohydrates, fat, and total energy intake were higher in men, which was consistent with previous studies [12]. Interestingly, the present study found gender differences in the impact of diet quality on mental health and health-related quality of life, and this discrepancy may be related to the quality of specific dietary components, such as fruits, vegetables, meat, fish, eggs, and legumes. 

A systematic literature review has provided evidence regarding diet quality, dietary patterns such as traditional Mediterranean diets and Western diets, and depression [17]. A cross-sectional study reported a significant association between a traditional diet, characterized by vegetables, fruit, meat, fish, and whole-grain foods, and a reduced likelihood of depressive and anxiety disorders [18]. Furthermore, another cohort study found that whole-food patterns rich in fruits, vegetables, and fish were associated with a decreased risk of depression, while processed food patterns rich in processed meat, chocolate, sweet deserts, fried food, refined cereals, and high-fat dairy products were associated with an increased risk of depression [19]. Findings from the Healthy Aging in Neighborhoods of Diversity across the Life Span (HANDLS) study consistently indicated that the diet quality measured by the HEI-2005 was associated with reduced odds of depressive symptoms among American adults [20]. Our study also used a Korean-specific eating index, and found a decreased risk of stress and depression after adjusting for confounding variables. These results are consistent with those of recent studies, which indicated that poor diet quality was associated with psychological distress, such as depressive symptoms, stress perception, and suicidal ideation in women, but not in men [10,11]. However, in one study, depressive symptoms and suicidal ideation were investigated using a single question [10], whereas another study was conducted only on middle-aged adults [11]. 

Nutritional status influences a person’s health-related quality of life by preventing malnutrition and promoting their health status [21]. The current study found that a higher-quality diet was significantly associated with a better quality of life, especially with regard to mobility and anxiety/depression problems. These results are consistent with those of several previous studies that have found a positive association between dietary patterns and quality of life, particularly in women [6,9,22]. A large cross-sectional study among North Americans reported that higher adherence to the Mediterranean diet was associated with a better quality of life, especially in the physical composite scale value [23]. Similarly, the Reach out to ENhancE Wellness (RENEW) trial found that diet quality had a positive association with physical quality of life outcomes such as better vitality and physical functioning [24]. The current findings are also supported by a longitudinal study conducted among older Australian adults, which reported that a healthier diet quality was associated with a better health-related quality of life, with additional associations with emotional well-being in women [6]. As the present study used a cross-sectional design, additional investigations are required to better understand these associations.

The relationship between diet, mental health, and health-related quality of life is complex and multidirectional, and it is not clear whether diet influences mental health and health-related quality of life, or whether mental health and quality of life are more influential, which may lead to changes in eating habits [4]. Although our understanding of these interactions is limited, possible mechanisms for this association may be explained by hormonal, inflammatory, and neural pathways [1,4]. A high glycemic index causes the secretion of counter-regulatory hormones and immune system activation due to high calories and saturated fat, and the interaction between the gut microbiome and the brain’s response may have detrimental effects on psychological well-being [1]. 

The strength of this study is that it evaluated the relationship between the KHEI, mental health, and health-related quality of life among a representative sample of Koreans. However, this study has some limitations. First, as it had a cross-sectional design we could not confirm whether this association represents a cause–effect relationship. Therefore, further longitudinal investigations or intervention research is needed to achieve greater levels of evidence for a causal relationship. Second, as the KHEI was calculated based on a single 24 h dietary recall instead of evaluating it based on the daily intake of the respondents, the scores might be underestimated or overestimated. Further studies on the daily intake of KHEI are required. Third, depression and health-related quality of life were assessed using standardized assessment tools, whereas stress perception was evaluated using a single question; thus, these factors may have been underestimated or overestimated. Finally, although we adjusted for potential confounding variables that could have affected mental health and health-related quality of life, additional factors may have influenced our results. Also, false-positive results were possibly obtained due to the multiple tests performed in this study.

## 5. Conclusions

The present study examined the association between diet quality evaluated using KHEI, mental health such as stress and depression, and the health-related quality of life among Korean men and women using the KNHANES. A high-quality diet was associated with a lower risk of stress, depression, and a better quality of life. Although these findings need to be confirmed by prospective longitudinal studies, they suggest that healthy eating behavior may improve mental health and quality of life. 

## Figures and Tables

**Table 1:** Demographic characteristics of the study population according to the tertiles of the KHEI score.

Variables	Men (*n* = 3004)							Women (*n* = 3819)						
	T1		T2		T3		*p*-Value	T1		T2		T3		*p*-Value
Age, years	39.9	±0.60	46.7	±0.60	50.1	±0.67	<0.001	41.1	±0.62	48.3	±0.60	52.5	±0.54	<0.001
BMI, kg/m^2^	24.5	±0.14	24.5	±0.13	24.0	±0.12	0.019	22.8	±0.13	23.2	±0.13	23.0	±0.11	0.073
Household income							0.016							0.386
Low	194	(15.2)	178	(13.3)	128	(11.2)		260	(17.4)	258	(16.6)	223	(15.8)	
Middle-low	257	(25.9)	232	(21.0)	251	(22.2)		315	(25.2)	324	(25.5)	320	(22.9)	
Middle-high	268	(28.9)	307	(32.4)	278	(29.5)		353	(28.8)	350	(27.9)	321	(27.8)	
High	272	(30.0)	305	(33.2)	334	(37.1)		332	(28.6)	367	(29.9)	396	(33.5)	
Education							<0.001							<0.001
≤Elementary school	117	(7.2)	181	(11.4)	136	(9.3)		269	(14.7)	353	(21.7)	302	(18.9)	
Middle school	92	(7.0)	106	(7.8)	91	(6.8)		84	(6.1)	130	(9.3)	165	(11.6)	
High school	395	(43.6)	324	(34.9)	336	(34.6)		414	(37.5)	404	(34.5)	364	(32.3)	
≥College	387	(42.2)	411	(45.8)	428	(49.3)		493	(41.6)	412	(34.5)	429	(37.2)	
Marital status							<0.001							<0.001
Married	653	(57.1)	838	(74.4)	860	(79.5)		961	(68.7)	1146	(83.6)	1176	(90.3)	
Unmarried	338	(42.9)	184	(25.6)	131	(20.5)		299	(31.3)	153	(16.4)	84	(9.7)	
Alcohol consumption							0.045							<0.001
<1 per month	280	(26.1)	288	(26.2)	308	(31.3)		640	(46.9)	759	(55.4)	788	(59.3)	
≥1 per month	711	(73.9)	734	(73.8)	683	(68.7)		620	(53.1)	540	(44.6)	472	(40.7)	
Smoking status							<0.001							<0.001
None	223	(25.6)	240	(25.1)	281	(31.3)		1031	(80.7)	1182	(89.7)	1186	(93.7)	
Ex-smoker	310	(27.2)	424	(37.6)	471	(42.3)		112	(8.9)	58	(4.6)	41	(3.2)	
Current smoker	458	(47.2)	358	(37.3)	239	(26.4)		117	(10.4)	59	(5.7)	33	(3.1)	
Aerobic physical activity							0.456							0.671
No	564	(53.6)	557	(50.5)	544	(51.1)		776	(56.9)	797	(58.3)	762	(58.7)	
Yes	427	(46.4)	465	(49.5)	447	(48.9)		484	(43.1)	502	(41.7)	498	(41.3)	
Total energy intake, kcal/day	2353.7	±38.0	2371.4	±30.5	2419.0	±25.8	0.271	1604.6	±27.6	1771.5	±23.2	1866.0	±16.9	<0.001

Data are expressed as mean ± standard error or number (%). Statistical differences between the groups were tested using linear regression analysis for continuous variables and chi-square tests for categorical variables. BMI, body mass index; KHEI, Korean healthy eating index.

**Table 2:** Total and components of the KHEI score according to the tertiles of the KHEI score.

Variables	Men (*n* = 3004)							Women (*n* = 3819)						
	T1		T2		T3		*p*-Value	T1		T2		T3		*p*-Value
Total KHEI score (0–100)	46.9	±0.28	62.4	±0.12	75.7	±0.23	<0.001	49.2	±0.28	65.6	±0.11	78.8	±0.18	<0.001
Adequacy														
Breakfast (0–10)	4.44	±0.17	7.39	±0.14	9.15	±0.09	<0.001	4.69	±0.14	7.53	±0.12	9.08	±0.08	<0.001
Mixed grains (0–5)	0.91	±0.06	1.91	±0.08	2.85	±0.08	<0.001	1.06	±0.05	1.93	±0.07	2.86	±0.07	<0.001
Total fruits (0–5)	0.59	±0.04	1.51	±0.08	3.03	±0.07	<0.001	1.17	±0.06	2.57	±0.07	3.92	±0.06	<0.001
Fresh fruits (0–5)	0.70	±0.06	1.72	±0.09	3.38	±0.08	<0.001	1.29	±0.07	2.85	±0.08	4.12	±0.07	<0.001
Total vegetable (0–5)	3.12	±0.06	3.87	±0.05	4.14	±0.05	<0.001	2.48	±0.05	3.32	±0.05	3.86	±0.04	<0.001
Vegetable, excluding kimchi and pickles (0–5)	2.62	±0.06	3.41	±0.06	3.81	±0.06	<0.001	2.22	±0.05	3.12	±0.05	3.79	±0.05	<0.001
Meat, fish, eggs, and legumes (0–10)	6.60	±0.14	7.58	±0.09	8.49	±0.08	<0.001	5.62	±0.12	7.16	±0.10	8.33	±0.07	<0.001
Milk and dairy (0–10)	1.92	±0.13	2.52	±0.16	4.91	±0.19	<0.001	2.40	±0.13	3.01	±0.15	5.08	±0.16	<0.001
Moderation														
Saturated fatty acid (0–10)	5.20	±0.19	7.87	±0.14	8.90	±0.09	<0.001	5.69	±0.17	7.81	±0.13	8.79	±0.09	<0.001
Sodium (0–10)	5.71	±0.13	5.36	±0.13	5.77	±0.11	0.036	7.79	±0.10	7.44	±0.10	7.71	±0.09	0.028
Sweets and beverages (0–10)	8.09	±0.12	9.38	±0.06	9.75	±0.05	<0.001	8.10	±0.11	9.51	±0.05	9.80	±0.03	<0.001
Balance														
Carbohydrate (0–5)	1.87	±0.08	2.84	±0.07	3.47	±0.06	<0.001	1.90	±0.07	2.55	±0.07	3.28	±0.06	<0.001
Fat (0–5)	2.68	±0.09	3.71	±0.06	4.25	±0.05	<0.001	2.62	±0.08	3.36	±0.07	4.22	±0.05	<0.001
Total energy (0–5)	2.46	±0.08	3.35	±0.07	3.78	±0.07	<0.001	2.15	±0.07	3.39	±0.07	3.94	±0.06	<0.001

Data are expressed as mean ± standard error. Differences between groups were tested using linear regression analysis.

**Table 3:** Mental health and health-related quality of life according to the tertiles of the KHEI score.

Variables	Men (*n* = 3004)							Women (*n* = 3819)						
	T1		T2		T3		*p*-Value	T1		T2		T3		*p*-Value
Stress perception														
No	705	(69.7)	802	(77.7)	819	(78.2)	<0.001	832	(64.9)	953	(72.8)	989	(78.5)	<0.001
Yes	286	(30.3)	220	(22.3)	172	(21.8)		428	(35.1)	346	(27.2)	271	(21.5)	
PHQ-9	2.14	±0.12	1.69	±0.10	1.73	±0.12	0.006	3.43	±0.14	2.74	±0.13	2.22	±0.10	<0.001
Depressive symptoms														
No (<10)	945	(95.8)	997	(98.1)	972	(97.9)	0.006	1148	(91.6)	1234	(94.8)	1215	(96.7)	<0.001
Yes (≥10)	46	(4.2)	25	(1.9)	19	(2.1)		112	(8.4)	65	(5.2)	45	(3.3)	
EQ-5D	0.97	±0.002	0.97	±0.002	0.97	±0.002	0.725	0.95	±0.003	0.95	±0.004	0.95	±0.003	0.190
Low EQ-5D index	141	(11.3)	160	(12.6)	142	(11.9)	0.661	291	(21.2)	302	(20.3)	283	(19.6)	0.622
Mobility														
No	900	(93.2)	917	(92.6)	887	(92.2)	0.691	1089	(88.8)	1090	(86.8)	1082	(89.0)	0.185
Some or extreme	91	(6.8)	105	(7.4)	104	(7.8)		171	(11.2)	209	(13.2)	178	(11.0)	
Self-care														
No	973	(98.8)	1003	(98.8)	962	(98.1)	0.304	1218	(97.5)	1255	(96.8)	1225	(98.1)	0.134
Some or extreme	18	(1.2)	19	(1.2)	29	(1.9)		42	(2.5)	44	(3.2)	35	(1.9)	
Usual activities														
No	945	(96.6)	976	(96.6)	944	(96.9)	0.926	1176	(95.0)	1204	(93.6)	1170	(94.6)	0.336
Some or extreme	46	(3.4)	46	(3.4)	47	(3.1)		84	(5.0)	95	(6.4)	90	(5.4)	
Pain/discomfort														
No	838	(86.1)	846	(84.8)	839	(86.5)	0.621	970	(79.0)	936	(73.7)	939	(76.6)	0.020
Some or extreme	153	(13.9)	176	(15.2)	152	(13.5)		290	(21.0)	363	(26.3)	321	(23.4)	
Anxiety/depression														
No	932	(95.2)	947	(93.8)	943	(94.7)	0.477	1089	(86.9)	1172	(90.8)	1137	(91.3)	0.004
Some or extreme	59	(4.8)	75	(6.2)	48	(5.3)		162	(13.1)	127	(9.2)	123	(8.7)	

Data are expressed as mean ± standard error or number (%). Low EQ-5D index was defined as the lowest quintile of the EQ-5D score. PHQ-9, Patient Health Questionnaire; EQ-5D, Euro Quality of Life Five-Dimensions.

**Table 4:** Association between the tertiles of the KHEI score, stress perception, depressive symptoms, and a low EQ-5D index.

Variables	Men (*n* = 3004)							Women (*n* = 3819)						
	T1		T2		T3		*p* for Trend	T1		T2		T3		*p* for Trend
Stress perception														
Unadjusted	1	(ref.)	0.662	(0.524–0.837) **	0.642	(0.496–0.831) **	0.001	1	(ref.)	0.691	(0.571–0.836) **	0.506	(0.419–0.611) **	<0.001
Model 1	1	(ref.)	0.737	(0.580–0.936) *	0.768	(0.587–1.004)	0.041	1	(ref.)	0.779	(0.640–0.950) *	0.619	(0.508–0.753) **	<0.001
Model 2	1	(ref.)	0.745	(0.585–0.949) *	0.807	(0.616–1.057)	0.090	1	(ref.)	0.815	(0.664–0.999) *	0.668	(0.541–0.823) **	<0.001
Depressive symptoms														
Unadjusted	1	(ref.)	0.432	(0.244–0.765) *	0.497	(0.265–0.933) *	0.021	1	(ref.)	0.606	(0.420–0.875) *	0.368	(0.233–0.582) **	<0.001
Model 1	1	(ref.)	0.425	(0.235–0.771) *	0.485	(0.241–0.979) *	0.033	1	(ref.)	0.569	(0.389–0.832) *	0.338	(0.209–0.546) **	<0.001
Model 2	1	(ref.)	0.519	(0.275–0.980) *	0.694	(0.332–1.447)	0.241	1	(ref.)	0.666	(0.458–0.968) *	0.464	(0.288–0.746) *	0.001
Low EQ-5D index														
Unadjusted	1	(ref.)	1.135	(0.854–1.510)	1.056	(0.795–1.402)	0.672	1	(ref.)	0.944	(0.762–1.169)	0.904	(0.740–1.104)	0.323
Model 1	1	(ref.)	0.868	(0.635–1.186)	0.714	(0.518–0.984) *	0.040	1	(ref.)	0.653	(0.518–0.823) **	0.538	(0.431–0.672) **	<0.001
Model 2	1	(ref.)	1.074	(0.772–1.495)	0.965	(0.686–1.357)	0.849	1	(ref.)	0.748	(0.588–0.951) *	0.722	(0.566–0.920) **	0.009

The data are expressed as odds ratios (95% confidence intervals).*: *p* < 0.05, **: *p* < 0.001. Model 1: adjusted for age and BMI. Model 2: Adjusted for age, BMI, income, education, marital status, smoking, alcohol consumption, aerobic physical activity, and total energy intake.

## Data Availability

The data are available from the KNHANES website.

## Supplementary Materials

The following supporting information can be downloaded at: https://www.mdpi.com/article/10.3390/nu15245111/s1, Table S1: Association between the tertiles of the KHEI score and the dimensional problem of EQ-5D; Table S2: Association between the tertiles of KHEI score, stress perception, depressive symptoms, and low EQ-5D index by age group.
