# Supplementary material for: Associations of Healthy Eating Behavior with Mental Health and Health-Related Quality of Life: Results from the Korean National Representative Survey
Source: Nutrients. 2023 Dec 14;15(24):5111. doi: 10.3390/nu15245111 (PMC10746005; doi:10.3390/nu15245111)
Supplement: Supplementary file 1 [file nutrients-15-05111-s001.zip › nutrients-2735928-supplementary.pdf]

Additional file

Table S1. Association between the tertiles of the KHEI score and the dimensional problem of EQ-5D

Variables	EQ-5D dimensions				
	Mobility	Self-care	Usual activities	Pain/discomfort	Anxiety/depression
	Model 2	Model 2	Model 2	Model 2	Model 2
**Men**					
T1	1	1	1	1	1
T2	0.863(0.570-1.307)	0.769(0.330-1.792)	0.916(0.527-1.589)	1.061(0.777-1.449)	1.460(0.919-2.319)
T3	0.848(0.550-1.308)	1.320(0.663-2.629)	0.826(0.450-1.517)	0.934(0.665-1.310)	1.299(0.787-2.146)
P for trend	0.461	0.408	0.537	0.710	0.263
**Women**					
T1	1	1	1	1	1
T2	0.769(0.564-1.050)	0.941(0.520-1.703)	0.967(0.657-1.424)	1.149(0.927-1.424)	0.683(0.487-0.958)*
T3	0.586(0.427-0.805)*	0.597(0.308-1.158)	0.827(0.563-1.213)	0.939(0.743-1.188)	0.663(0.484-0.910)*
P for trend	0.001	0.130	0.322	0.603	0.010

The data are expressed as odds ratios (95% confidence intervals).

*$P < 0.05$, **$P < 0.01$.

Model 2: Adjusted for age, BMI, income, education, marital status, smoking, alcohol consumption, aerobic physical activity, and total energy intake.

Table S2. Association between the tertiles of KHEI score, stress perception, depressive symptoms, and low EQ-5D index by age group

Variables	Stress perception	Depressive symptoms	Low EQ-5D index
	Model 2	Model 2	Model 2
**Men**			
**19–64 (n = 2,272)**			
T1	1	1	1
T2	0.804 (0.626-1.032)	0.613 (0.301-1.248)	1.093 (0.753-1.588)
T3	0.766 (0.579-1.013)	0.657 (0.273-1.583)	0.912 (0.583-1.427)
P for trend	0.056	0.288	0.706
**65–80 (n = 732)**			
T1	1	1	1
T2	0.935 (0.519-1.684)	0.559 (0.171-1.832)	1.107 (0.648-1.891)
T3	0.716 (0.365-1.404)	1.227 (0.364-4.133)	0.971 (0.560-1.681)
P for trend	0.323	0.812	0.929
**Women**			
**19–64 (n = 2,917)**			
T1	1	1	1
T2	0.824 (0.658-1.031)	0.711 (0.464-1.089)	0.808 (0.595-1.095)
T3	0.641 (0.507-0.809)**	0.391 (0.209-0.732)**	0.717 (0.522-0.984)*
P for trend	<0.001	0.002	0.039
**65–80 (n = 902)**			

T1	1	1	1
T2	0.883 (0.579-1.345)	0.737 (0.362-1.500)	0.900 (0.587-1.379)
T3	0.609 (0.356-1.040)	0.517 (0.235-1.138)	0.773 (0.517-1.156)
P for trend	0.068	0.087	0.212

The data are expressed as odds ratios (95% confidence intervals).

$^{*}P < 0.05$, $^{**}P < 0.01$.

Model 2: Adjusted for age, BMI, income, education, marital status, smoking, alcohol consumption, aerobic physical activity, and total energy intake.
